# College and Hospital Notes

**Published:** 1900-08

**Authors:** 


### COLLEGE AND HOSPITAL NOTES.

The Kentucky School of Medicine celebrated its golden anniversary at its annual commencement held at Louisville, June 26, 1900. A program of great interest had been prepared, including an elaborate musical schedule, the conferring of the degrees upon the graduating classes by Hon. John H. Leathers, president of the board of regents, the announcement of honors by Dr. William H. Wathen, dean of the faculty. The event of the evening was the doctorate address delivered by Professor Roswell Park, of Buffalo. Dr. Park, always an interesting speaker, was unusually so on this occasion and his address abounded with graceful rhetoric and eloquent periods. The ceremonies were every way worthy of the occasion, upon which the Kentucky School of Medicine is to be congratulated.

---

THE Albany Hospital graduated the first class from its training school for nurses, May 10, 1900. The occasion was one of great interest to the friends of the hospital, which is one of the oldest and most famous in the country. Thirteen young women who had taken the three years' course of hospital training under the direction of the superintendent of the training school, Miss Emily MacDonnell, received diplomas.

Dr. A. Vander Veer, Surgeon-in-Chief of the hospital, delivered an address to the class which was frought with interest and eloquence, and Mrs. Hunter Robb, of Cleveland, O., also addressed the assemblage. The diplomas were conferred by Judge William L. Learned, and after the ceremonies the hospital was thrown open to guests, members of the medical staff and nurses acting as a reception committee, and in the Nurses' Home an elaborate luncheon was served.

